# Mitochondrial dysfunction in *MED13* variant-associated disease: a case of infantile spasms, cardiomyopathy and hepatomegaly

**DOI:** 10.1038/s41439-025-00327-x

**Published:** 2025-10-23

**Authors:** Mizuki Harada, Takanori Onuki, Hiromi Nyuzuki, Hisato Suzuki, Kozue Ito, Go Hasegawa, Hideki Hayashi, Mari Tada, Toshiki Takenouchi, Kei Murayama, Hiroshi Suzuki

**Affiliations:** 1 Department of Pediatrics, Uonuma Kikan Hospital, Niigata, Japan; 2 Department of Pediatrics, Division of Neonatology, Niigata University School of Medicine, Niigata, Japan; 3 Department of Pediatrics, Division of Homeostatic Regulation and Development, Niigata University Graduate School of Medical and Dental Sciences, Niigata, Japan; 4 Center for Medical Genetics, Niigata University Medical and Dental Hospital, Niigata, Japan; 5 Center for Medical Genetics, Keio University School of Medicine, Tokyo, Japan; 6 Department of Pathology, Uonuma Kikan Hospital, Niigata, Japan; 7 Department of Pathology, Uonuma Institute of Community Medicine, Niigata University Medical and Dental Hospital, Niigata, Japan; 8 Department of Pathology, Brain Research Institute, Niigata University, Niigata, Japan; 9 Department of Pediatric Neurology, Okayama University Graduate School of Medicine, Dentistry and Pharmaceutical Sciences, Okayama, Japan; 10 Department of Pediatrics, and Diagnostics and Therapeutic of Intractable Diseases, Intractable Disease Research Center, Graduate School of Medicine, Juntendo University, Tokyo, Japan; 11 Uonuma Institute of Community Medicine, Niigata University Medical and Dental Hospital, Niigata, Japan

**Keywords:** Gene regulation, Mutation

## Abstract

Here we report a de novo heterozygous *MED13* variant (c.2503C>T, p.Pro835Ser) in an infant presenting with infantile spasms, hypertrophic cardiomyopathy and hepatomegaly. Autopsy revealed mitochondrial abnormalities in cardiac and hepatic tissues, with reduced respiratory chain complex activity. This is the first case report linking a *MED13* variant to systemic mitochondrial dysfunction, suggesting a novel pathogenic mechanism.

## Introduction

Mediator complex subunit 13 (MED13) is a component of the regulatory portion of the mediator complex, which comprises over 30 subunits^1^. The mediator complex functions as a bridge between DNA-binding transcription factors and RNA polymerase II. Among these subunits, MED13 plays a key role in the MED–CDK8 interaction, thereby contributing to the regulation of gene expression^2^. Heterozygous variants in *MED13* genes have been associated with neurodevelopmental disorders of varying severity^3–7^. The underlying pathogenesis remains poorly understood. We report a Japanese male infant with a *MED13* variant who presented with infantile spasms, cardiomyopathy and hepatomegaly in whom autopsy findings revealed mitochondrial abnormalities.

**Fig. 1:**
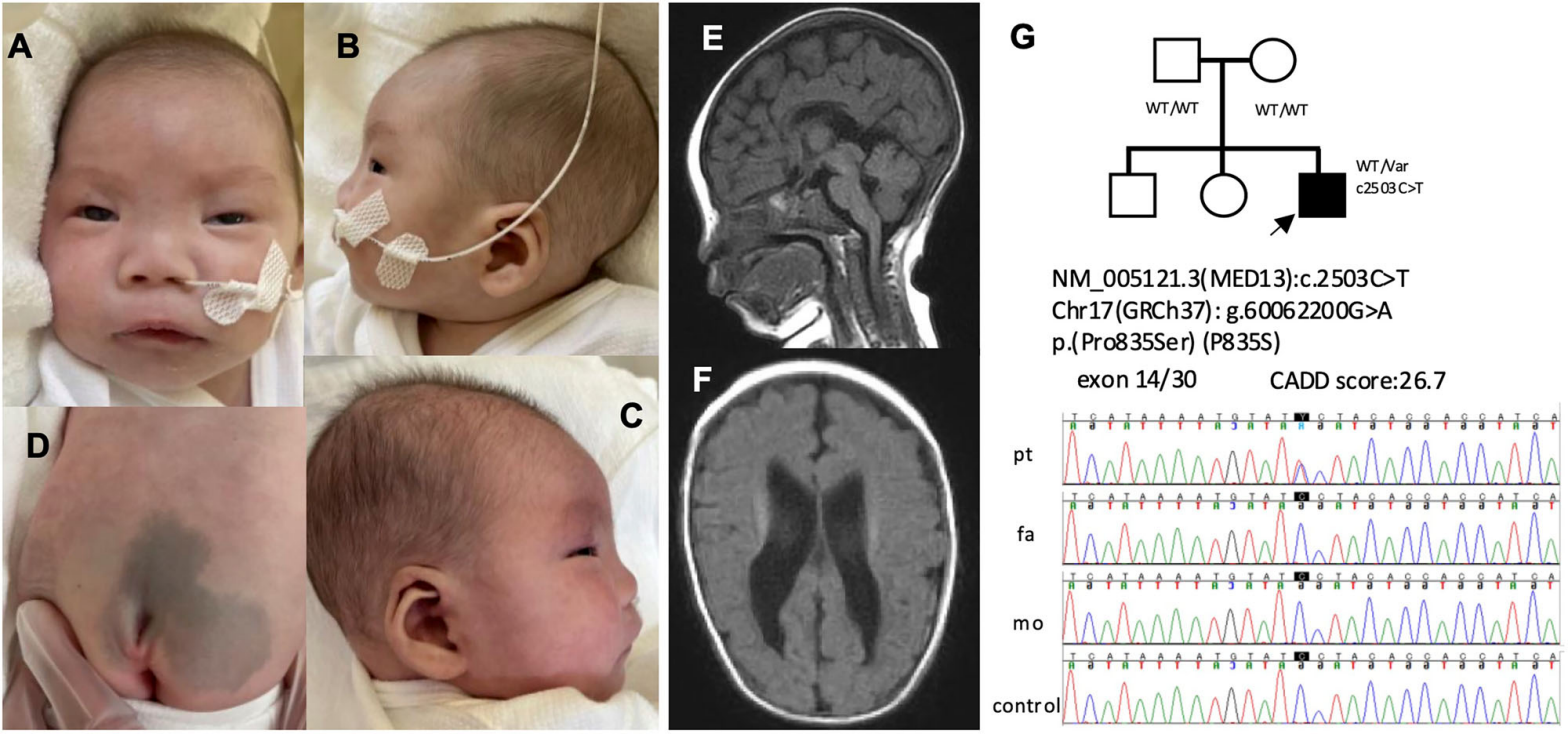
Clinical and Genetic Findings of the Patient. **A**–**C** Facial appearance at 3 months was marked by hypertelorism, low-set ears, a broad nasal root and thin upper lip. **D** Sacral dimple in the lumbosacral region. **E**, **F** Brain MRI (T1-weighted images) at 4 months revealed corpus callosum hypoplasia and bilateral ventricular enlargement. **G** Family pedigree of the index case. Sanger sequencing revealed a de novo missense variant of *MED13* in the patient. WT, wild type; Var, variant; pt, patient; fa, father; mo, mother.

The patient was the third child of healthy, nonconsanguineous parents. Fetal growth restriction and bilateral ventricular enlargement were noted during pregnancy. He was delivered via emergency cesarian section at 33 weeks 4 days of gestation due to fetal growth failure. At birth, his weight was 1313 g (−2.68 SD), height was 40.0 cm (−1.49 SD) and head circumference was 28.5 cm (−1.13 SD). Initial neonatal care included noninvasive positive-pressure ventilation for 1 month. Dysmorphic features included hypertelorism, low-set ears, a broad nasal root and a sacral dimple (Fig. 1A–D). At 2 months of age, tonic seizures developed, which deformed into epileptic spasms by 4 months. Brain magnetic resonance imaging (MRI) revealed a hypoplastic corpus callosum and bilateral ventricular enlargement (Fig. 1E, F). Electroencephalography findings demonstrated a suppression burst pattern, consistent with Ohtahara syndrome. While treatment with sodium valproate, phenobarbital, high-dose steroids and potassium bromide was ineffective, seizure control was finally achieved with vigabatrin. At 3 months, blood lactate and pyruvate levels were 6.34 and 0.18 mmol/l, respectively (L/P ratio 35.2), while cerebrospinal fluid lactate and pyruvate were 1.54 and 0.07 mmol/l, respectively. The patient also had bilateral optic nerve atrophy, macular hypoplasia and severe bilateral hearing loss. Echocardiography demonstrated hypertrophic cardiomyopathy first noted at 2 months. Left ventricular outflow tract obstruction gradually progressed, for which a beta-blocker was initiated at 7 months. He was first discharged at 7 months. Severe developmental delay was evident; he lacked head control or eye tracking, required tube feeding and showed minimal weight gain. Hepatomegaly was noted before discharge, although liver function was preserved. Computed tomography scan conducted at 9 months indicated an enlarged liver with heterogeneous parenchymal density. He was hospitalized at 9 months for acute respiratory failure due to infection. Although successfully extubated after 2 weeks of mechanical ventilation, he continued to require noninvasive positive-pressure ventilation support. Marked hypertonia impeded feeding, which improved with tizanidine, and the initiation of home high-flow therapy enabled discharge at 11 months. At 17 months, he died from aspiration-induced airway obstruction.

Written informed consent was obtained from the parents for testing whole exome sequencing of the patient and both parents. Genetic testing identified a de novo heterozygous missense variant of *MED13* in the patient (NC_000017.11(NM_005121.3):c.2503C>T p.(Pro835Ser)) (Fig. 1G). The variant is classified as pathogenic according to the American College of Medical Genetics and Genomics and the Association for Molecular Pathology guidelines (PS1 + PS2 + PM2 + PP3). No pathogenic mitochondrial DNA variants were identified.

**Fig. 2:**
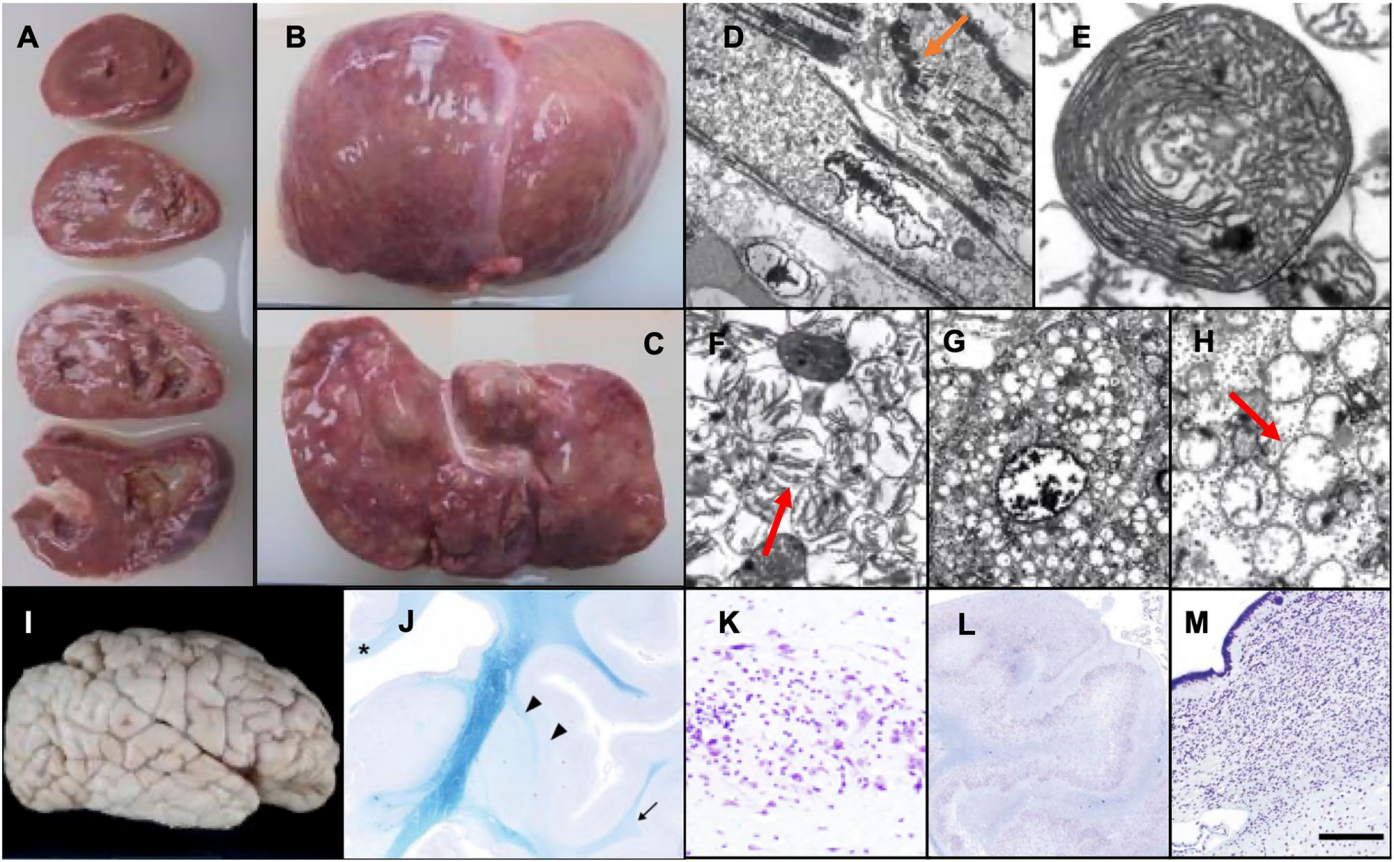
Autopsy Pathological Findings of the Heart, Liver and Brain. **A**–**C** Gross examination of the heart and liver. Myocardial hypertrophy and nodular hepatomegaly. **D**–**H** Electron microscopic findings of myocardial cells (**D**–**F**) and hepatocytes (**G** and **H**): a marked increase in myocardial mitochondria separating myofibrils (orange arrow, ×2000) (**D**); abnormal mitochondrial cristae formation resembling fingerprints (×25,000) (**E**); myocardial mitochondrial vacuolization (red arrow, ×10,000) (**F**); a marked increase of mitochondria within hepatocytes (×3000) (**G**); hepatic mitochondrial vacuolization (red arrows), ×10,000) (**H**). **I** Lateral view of the right cerebrum. Atypical gyration in the parietal lobe and a deep sylvian fissure. **J** Coronal section of the cerebrum. Diffuse white matter hypoplasia accentuated in the corpus callosum (asterisk), external and extreme capsules (arrowheads) and temporal lobe (arrow). **K** Immature cell foci with enclosed neurons in the amygdaloid nucleus. **L** Coronal section of the hippocampus. Hippocampal malrotation with excessive folding. **M** Persistence of germinal cells in the subventricular zone. In **J**–**M** Klüver–Barrera staining was applied. Scale bar, 100 μm (**K**) and 200 μm (**M**).

Autopsy was performed with written parental consent. Gross examination revealed hypertrophic myocardium and an enlarged, nodular liver (Fig. 2A–C). Electron microscopy revealed abnormal mitochondrial morphology in cardiac and hepatic tissues (Fig. 2D–H). Brain examination revealed atypical gyration and diffuse white matter hypoplasia in the cerebrum, hippocampal malrotation and residual germinal cells in the subventricular zone (Fig. 2I–M). Mitochondrial respiratory chain enzyme activity was analyzed in liver, kidney, cardiac and skeletal muscle samples. The complex I citrate synthase activity ratio (CS ratio) was markedly reduced by 26.1% in the liver and 24.6% in the cardiac muscle. Given that a CS ratio of <20% meets the major diagnostic criterion and <30% the minor criterion described previously^8,9^, these reductions represent a substantial impairment of mitochondrial enzymatic activity.

*MED13* variants are known to be associated with mild neurodevelopmental delays. Furthermore, more severe phenotypes have recently been reported. Trivisano et al. described a case with infantile epilepsy and a missense variant (p.Tyr834Cys)^6^, which exhibited corpus callosum hypoplasia and encephalopathy that mirror our case. Tolmacheva et al. reported a case with the same variant as the present case (p.Pro835Ser) with severe congenital heart conditions, bronchopulmonary dysplasia and ileal atresia, leading to early mortality^7^. Although dysmorphic facial features, growth restriction, ocular abnormalities and MRI findings resemble our case, hepatomegaly and hypertrophic cardiomyopathy distinguished our case, suggesting phenotypic variability within the same variant. In addition, autopsy findings revealed abnormal mitochondrial features in the affected organs, representing the first documented case suggesting mitochondrial involvement in a *MED13* variant. Elevated blood lactate and L/P ratio during life also implied potential mitochondrial dysfunction, although recurrent seizures at the time of the sampling may have contributed to the results.

MED13 is a key component of the CDK8 kinase module within the mediator complex and serves as a nuclear anchor of cyclin C^10^. Experimental models support a role in mitochondrial dynamics. In *Drosophila*, Cdk8 loss in neurons and muscle showed elongated mitochondria, impaired mitochondria fission with concomitant reductions in ATP synthesis and led to impaired wing posture and reduced lifespan^11^. In fibroblasts from a patient with a *MED13* variant (p.L830R), Pijuan et al. observed elongated mitochondrial networks, reduced fragmentation and a significant decrease in mitochondrial mass^12^. In yeast, loss of Med13p function releases cyclin C into the cytoplasm, inducing extensive mitochondrial fission and hypersensitivity to oxidative-stress-induced programmed cell death^13^. These findings suggest that MED13 may influence mitochondrial dynamics and play a role in cellular stress response pathways involving cyclin C and CDK8.

The mechanisms underlying the phenotypic variability of *MED13* variants remain unclear. However, the autopsy findings and mitochondrial respiratory chain enzyme analysis in our case suggest systemic mitochondrial dysfunction as a potential contributor to disease pathogenesis. Further studies are needed to elucidate the precise role of *MED13* in mitochondrial dynamics and its implications for disease severity.

## HGV Database

The relevant data from this Data Report are hosted at the Human Genome Variation Database at 10.6084/m9.figshare.hgv.3547.
